# Thrap3 promotes nonalcoholic fatty liver disease by suppressing AMPK-mediated autophagy

**DOI:** 10.1038/s12276-023-01047-4

**Published:** 2023-08-01

**Authors:** Hyun-Jun Jang, Yo Han Lee, Tam Dao, Yunju Jo, Keon Woo Khim, Hye-jin Eom, Ju Eun Lee, Yi Jin Song, Sun Sil Choi, Kieun Park, Haneul Ji, Young Chan Chae, Kyungjae Myung, Hongtae Kim, Dongryeol Ryu, Neung Hwa Park, Sung Ho Park, Jang Hyun Choi

**Affiliations:** 1 Department of Biological Sciences, Ulsan National Institute of Science and Technology (UNIST), Ulsan, 44919 Republic of Korea; 2 Herbal Medicine Resources Research Center, Korea Institute of Oriental Medicine, Naju, 58245 Republic of Korea; 3 Department of Molecular Cell Biology, Sungkyunkwan University (SKKU) School of Medicine, Suwon, 16419 Republic of Korea; 4 Center for Genomic Integrity, Institute for Basic Science, Ulsan, 44919 Republic of Korea; 5 Department of Internal Medicine, University of Ulsan College of Medicine, Ulsan University Hospital, Ulsan, 44033 Republic of Korea

**Keywords:** Metabolic disorders, Autophagy

## Abstract

Autophagy functions in cellular quality control and metabolic regulation. Dysregulation of autophagy is one of the major pathogenic factors contributing to the progression of nonalcoholic fatty liver disease (NAFLD). Autophagy is involved in the breakdown of intracellular lipids and the maintenance of healthy mitochondria in NAFLD. However, the mechanisms underlying autophagy dysregulation in NAFLD remain unclear. Here, we demonstrate that the hepatic expression level of Thrap3 was significantly increased in NAFLD conditions. Liver-specific Thrap3 knockout improved lipid accumulation and metabolic properties in a high-fat diet (HFD)-induced NAFLD model. Furthermore, Thrap3 deficiency enhanced autophagy and mitochondrial function. Interestingly, Thrap3 knockout increased the cytosolic translocation of AMPK from the nucleus and enhanced its activation through physical interaction. The translocation of AMPK was regulated by direct binding with AMPK and the C-terminal domain of Thrap3. Our results indicate a role for Thrap3 in NAFLD progression and suggest that Thrap3 is a potential target for NAFLD treatment.

## Liver disease: Poor cellular maintenance fuels fatty liver

A protein that interferes with a critical cellular housekeeping mechanism offers a promising drug target for non-alcoholic fatty liver disease (NAFLD), a condition that can lead to liver failure. Jang Hyun Choi of the Ulsan National Institute of Science and Technology, South Korea, and colleagues previously demonstrated that a protein called Thrap3 contributes to obesity-related insulin resistance. They have now investigated Thrap3 function in the liver and found that levels were significantly elevated in tissues from NAFLD patients. The researchers demonstrated that they could reduce NAFLD-associated lipid accumulation by using genetic techniques to shut down Thrap3 expression in liver cells. Thrap3 was found to inhibit autophagy, a cellular process for clearing out defective proteins and organelles. Impaired autophagy is a known contributor to NAFLD, and the authors hypothesize that Thrap3 inhibition could protect against this impairment.

## Introduction

Obesity can lead to various organ and vascular complications, including nonalcoholic fatty liver disease (NAFLD)^1^. NAFLD is the most common chronic liver disease and is characterized by hepatic fat accumulation, inflammation and oxidative stress. It is considered a hepatic manifestation of metabolic syndrome, ranging from benign steatosis to severe nonalcoholic steatohepatitis (NASH), potentially further damaging organs^2^. Sustained elevation of neutral lipids, mostly triglycerides (TGs), and accumulation in hepatocyte lipid droplets initiates the early onset of NAFLD. Fatty acids originating from different sources, including dietary lipid intake, de novo lipogenesis and adipose tissue lipolysis, contribute to the pathogenesis of steatosis^3^. Despite the increasing prevalence of NAFLD, there are currently no effective therapies for NAFLD. Lifestyle interventions such as exercise and weight loss are only helpful to some extent. Therefore, there is a need to discover new therapeutic targets and approaches for treating NAFLD.

Autophagy is a quality control process for organelle health, during which damaged intracellular components, including lipid droplets and mitochondria are engulfed and degraded. Impaired autophagy is associated with the development and progression of hepatic steatosis and NAFLD^4^. Moreover, the enhancement of autophagy by genetic modification or pharmacological intervention alleviates liver steatosis in NAFLD disease models^5,6^. Therefore, autophagy has gained attention as a promising therapeutic target for NAFLD. Autophagy responds to nutrient status through AMP-activated protein kinase (AMPK) and mammalian target of rapamycin complex 1 (mTORC1)^7^. In particular, AMPK promotes autophagy directly by phosphorylating autophagy-related proteins, such as ULK1 and mTORC1, or indirectly by increasing the expression of autophagy-related genes downstream of transcription factors, such as FOXO3, TFEB and BRD4. AMPK activity is reduced by inflammation, obesity, diabetes and NAFLD. Several preclinical and clinical studies have highlighted that AMPK influences NAFLD and that its activators reduce NAFLD by enhancing autophagy^8^. However, the underlying mechanism for the dysregulation of the AMPK/autophagy axis in NAFLD remains unclear.

Thyroid hormone receptor-associated protein 3 (Thrap3) is a pre-mRNA splicing factor that triggers mRNA decay in the spliceosome of nuclear speckles. Thrap3 also acts as a transcriptional coregulator. In a previous report, we showed that Thrap3 interacts with PPARγ and controls diabetic gene programming in adipose tissues. Reduction in Thrap3 levels in fat tissue with antisense oligonucleotides (ASOs) improves glucose homeostasis in obese mice^9^. The function of Thrap3 in the liver remains unclear, although reduced hepatic expression of Thrap3 could affect metabolic homeostasis in ASO experiments. In the present study, we demonstrated the role of Thrap3 in the liver, particularly in NAFLD. Our data suggest a novel mechanism by which Thrap3 suppresses autophagy by blocking the translocation and activation of AMPK. These findings establish Thrap3 as a potential therapeutic target for preventing and treating NAFLD by regulating the AMPK/autophagy axis.

## Materials and methods

### Mouse primary hepatocyte isolation

Primary hepatocyte isolation was performed as described previously^10^. Briefly, mice were anesthetized with isoflurane, and a 24-gauge needle was inserted into the portal vein. Then, the inferior vena cava was cut, and the mouse liver was perfused sequentially with solution I (142 mM NaCl, 67 mM KCl, 10 mM HEPES and 2.5 mM EGTA) and solution II (66.7 mM NaCl, 6.7 mM KCl, 50 mM HEPES, 4.8 mM CaCl_2_·2H_2_O and 0.02% Type IV collagenase (Sigma‒Aldrich, St. Louis, MO)). After perfusion, the liver was dissected and disrupted in M199 with EBSS (M199/EBSS) medium. The obtained cells were filtered through a 70 μm cell strainer, and the cell suspension was spun at 50 × g for 5 min at 4 °C. The supernatant was gently aspirated, and the cells were resuspended in M199/EBSS and gently loaded onto an equal volume of Percoll solution (27% Percoll). The cell suspension was spun at 200 × g for 2 min at 4 °C, and the pellet was washed once with M199/EBSS. After viable cells were counted with trypan blue, the isolated hepatocytes were seeded in M199/EBSS supplemented with 10% FBS and 1% penicillin/streptomycin.

### Cell culture

Human liver HepG2 cells were purchased from American Type Culture Collection (ATCC, Manassas, VA) and cultured in Dulbecco’s Modified Eagle’s Medium (DMEM) containing 10% fetal bovine serum (Gibco, BRL, Grand Island, NY) and 1% penicillin/streptomycin (Thermo Fisher Scientific, Waltham, MA). siRNA was purchased from GenePharma (Shanghai, China). The siRNAs used in this study are listed in Supplementary Table 1. HepG2 cells and primary hepatocytes were transfected with siRNA or GFP-tagged or HA-tagged deletion mutants of the Thrap3 plasmid^9^, pEGFP-LC3 (#21073, Addgene)^11^ using Lipofectamine^TM^ RNAiMAX transfection reagent or Lipofectamine^TM^ 2000 (Thermo Fisher Scientific, Waltham, MA) according to the manufacturer’s instructions. The following experiments were performed 48 h after transfection, and for intracellular lipid accumulation, cells were cultured in medium supplemented with 1 mmol/L FFA (oleic acid and palmitic acid, ratio 2:1), 10 nmol/L bafilomycin A1 (19–148, Sigma‒Aldrich, St. Louis, MO) or 10 μmol/L Compound C (171260, Sigma‒Aldrich, St. Louis, MO) for 24 h, and then the cells were harvested for further analysis.

### Human patients

Human liver tissue samples from 265 patients were provided by Ulsan University Hospital, Ulsan, Republic of Korea. The process was officially approved by the Institutional Review Board at the Ulsan University Hospital (UUH 2015-12-018). The human liver samples used in this study were from hepatocellular carcinoma (HCC) liver resection patients. All samples were taken from nontumor parts of the resected tissue and stratified into three main groups based on their histological fatty change profiles: 197 grade 0 normal liver samples, 62 grade 1 mild fatty change samples (defined by 5 to 10% of hepatocytes exhibiting cytoplasmic lipid droplets) and 6 grade 2 severe fatty change samples (defined by a proportion of hepatocytes containing lipid droplets exceeding 10%). Although the samples from nontumor parts have limitations in completely excluding the effect of HCC, histologically normal tissue adjacent to the tumor has obvious distinctions in global transcriptome patterns for the tumor and can be used as a normal control^12^.

### Analysis of the publicly available human hepatic transcriptome

From the NCBI GEO database, we collected human hepatic transcriptomic datasets spanning obesity or nonalcoholic fatty liver disease (GSE15653, GSE135251 and GSE130970)^13–16^. The data from GSE135251 were processed as described previously^17^. Briefly, the data were normalized using the trimmed mean of M-values (TMM) normalization method in EdgeR (https://bioconductor.org/packages/release/bioc/html/edgeR.html). *THRAP3* expression was compared between healthy controls and patients with obesity or NAFLD and across the NAS score.

### RNA-seq and bioinformatic analysis

RNA-seq was performed on two individual samples from liver NCD-fed wild-type and Thrap3 LKO mice. Total RNA was isolated using the RNeasy mini kit (Qiagen, Hilden, Germany) according to the manufacturer’s instructions. A total amount of 1 μg RNA per sample was used as input material for the RNA sample preparations. The quality and quantity of the total RNA were evaluated using an Agilent 2100 Bioanalyzer RNA kit (Agilent Technologies, Inc., Santa Clara, CA). Sequencing libraries were generated using an Illumina TruSeqStranded mRNA Sample Preparation kit (Illumina, Inc., San Diego, CA) following the manufacturer’s recommendations. Library quality and size were assessed on the Agilent Bioanalyzer 2100 system (Agilent Technologies, Inc., Santa Clara, CA). The library preparations were sequenced on NextSeq500 sequencers (Illumina, Inc., San Diego, CA), and paired-end 75 bp plus single 8 bp index reads were generated. After quality filtering according to the Illumina pipeline, paired-end reads were mapped to the reference genome (mm10 assembly) using STAR aligner version 2.7.3a with default parameters. Differentially expressed genes (DEGs) were identified using DESeq2 1.30.0. Read counts for DESeq2 analysis were obtained using featureCounts v2.0.0. After eliminating absent and low features (zero or <1CPM), the raw counts were normalized using DESeq2, followed by differential expression analysis. The KEGG enriched pathway was determined according to ErichR, the web-based software for gene set enrichment analysis.

### Immunoblotting and immunoprecipitation

Supernatants containing protein contents were determined by Bradford Protein Assay (Bio-Rad Laboratories, Hercules, CA). For immunoprecipitation, cleared cell extracts were incubated with specific antibodies overnight. Then, the protein-antibody samples were mixed with protein A/G agarose beads for 2 h and precipitated. Proteins were immunoblotted with anti-Thrap3 (sc-133249, Santa Cruz Biotechnology, Dallas, TX), anti-phospho-Akt (#9271, Cell Signaling Technology, Danvers, MA), anti-Akt (sc-5298, Santa Cruz Biotechnology, Dallas, TX), anti-LC3 (#2775, Cell Signaling Technology, Danvers, MA), anti-p62 (#5114, Cell Signaling Technology, Danvers, MA), anti-phospho-ULK1 (#12753, Cell Signaling Technology, Danvers, MA), anti-ULK1 (#8054, Cell Signaling Technology, Danvers, MA), anti-phospho-AMPK (#2535, Cell Signaling Technology, Danvers, MA), anti-AMPK (#2532, Cell Signaling Technology, Danvers, MA), anti-phospho-ACC (#11818, Cell Signaling Technology, Danvers, MA), anti-ACC (#3662, Cell Signaling Technology, Danvers, MA), anti-FLAG (F4042, Sigma‒Aldrich, St. Louis, MO), anti-HA (sc-7392, Santa Cruz Biotechnology, Dallas, TX), anti-Tubulin (MA5-16308, Thermo Fisher Scientific, Waltham, MA), anti-Lamin B1 (sc-374015, Santa Cruz Biotechnology, Dallas, TX), anti-β-actin (GTX629630, Genetex, Irvine, CA), anti-SDHB (ab14714, Abcam, Cambridge, UK), anti-UQCRC2 (ab14745, Abcam, Cambridge, UK), anti-ATP5A (ab14748, Abcam, Cambridge, UK), anti-PINK1 (BC100-494, Novus Biologicals, Englewood, CO) and anti-HSP90 (#4877, Cell Signaling Technology, Danvers, MA).

### Immunofluorescence, fluorescence and confocal microscopy assays

Primary hepatocytes were seeded on confocal chamber slides. Cells were washed with PBS and fixed in 4% paraformaldehyde for 15 min at RT. The fixed cells were permeabilized with 0.5% Triton X-100, blocked with blocking buffer for 1 h, and incubated with anti-AMPK antibody overnight at 4 °C. After washing, the cells were incubated with Alexa Fluor 594 secondary antibodies for 1 h and stained with DNA-specific fluorescent Hoechst dye. PBS-washed cells were visualized with an LSM 880 confocal laser scanning microscope (Carl Zeiss, Germany).

For the GFP-LC3 assay, primary hepatocytes were transfected with pEGFP-LC3. Cells were fixed with 4% paraformaldehyde for 15 min at RT and coverslipped. MitoTracker™ Red CMXRos and Hoechst dye were used for mitochondrial and nuclear staining according to the instructions. GFP-LC3 and MitoTracker were visualized with a confocal laser scanning microscope LSM 880 (Carl Zeiss, Germany). Representative cell images were randomly selected for analysis, and the numbers of GFP-LC3 puncta per cell were calculated.

### Quantitative PCR

Total mRNA was isolated using TRIzol reagent (Thermo Fisher Scientific, Waltham, MA). RNA reverse transcription was performed using an ABI Reverse Transcription Kit (Thermo Fisher Scientific, Waltham, MA). Quantitative PCR was performed with a 7900HT Fast Real-Time PCR System (Life Technologies, Carlsbad, CA) following the manufacturer’s instructions. Relative mRNA expression levels of each gene were normalized to the expression level of the TATA-binding protein TBP. The mtDNA copy number was evaluated based on the ratio of mtDNA to nuclear DNA by quantitative PCR. The mtDNA was quantified based on the mitochondrial gene *Polg*. The nuclear DNA was quantified based on the nuclear DNA gene *Actb*. The primer pairs used in this study are listed in Supplementary Table 2.

### Cellular oxygen consumption rate (OCR)

OCR was analyzed by a Seahorse XF24 extracellular flux analyzer (Seahorse Bioscience, North Billerica, MA) following the manufacturer’s instructions. The results were normalized to the protein quantity of each corresponding well.

### Mice

All animal experiments were performed according to procedures approved by the Ulsan National Institute of Science and Technology’s Institutional Animal Care and Use Committee (UNISTIACUC-19-04). Thrap3 conditional mice were generated by breeding C57BL/6N-Atm1Brd *Thrap3*^tm1a(KOMP)Wtsi^/MbpMmucd mice (Mutant Mouse Regional Resource Centers (MMRRC), Stock #: 050053-UCD) with *Flp* mice. Liver-specific Thrap3 knockout mice were generated by crossing conditional floxed Thrap3 mice with B6 mice. Cg-Tg(alb-cre)21Mgn/J mice (Jackson Laboratory, Bar Harbor, ME). The mice were housed in specific pathogen–free conditions under a 12 h light/dark cycle at a temperature of 21 °C and allowed free access to normal chow diet (A03, Scientific Animal Food & Engineering, Augy, France) and water. Seven-week-old male mice were fed a HFD (60% kcal fat, D12492, Research Diets Inc., New Brunswick, NJ) for 12 weeks or a MCD (A02082002BR, Research Diets Inc., New Brunswick, NJ, USA) for 4 weeks.

### Metabolic analysis

Mice were fasted overnight (18 h) before intraperitoneal injection of D-glucose (2 g/kg body weight) for the glucose tolerance test. For the insulin tolerance test, mice were fasted for 4 h before intraperitoneal injection of insulin (0.75 U/kg body weight). Every glucose level was examined with tail vein blood at the indicated intervals after injection using a glucometer. To analyze metabolic parameters, TG (10010303, Cayman Chemical, Ann Arbor, MI), cholesterol (K603, BioVision Inc.), FFA (BM-FFA-100, BioMax, Republic of Korea), insulin (90080, Crystal Chem, Elk Grove Village, IL), ALT (K752, Biovision Inc., Milpitas, CA) and AST (K753, BioVision Inc.) were determined. Body weight was measured weekly. The body composition of the mice was measured using a quantitative nuclear resonance system EchoMRI100V (Echo Medical Systems, Houston, TX). Oxygen consumption, food intake and locomotor activity were measured using a Comprehensive Lab Animal Monitoring System (CLAMS; Columbus Instruments, Columbus, OH, USA) as described previously^18^.

### Histological analysis

Mouse liver tissues were isolated and immediately fixed with 4% formalin. Lipid droplets were analyzed by H&E staining and Oil Red O staining. Mayer’s hematoxylin was used as a counterstain for every slide. Liver fibrosis was further determined by Sirius red staining. Images were obtained with an Olympus BX53 microscope and DP26 camera. NAS and fibrosis scores were measured by a pathologist according to the NASH Clinical Research Network scoring system^19^. For statistical analysis of the fibrosis score, each grade (0~4) was calculated by giving a score from 1 to 6 points and presented as grade on the graph.

### Electron microscopy

Samples were fixed with 2% glutaraldehyde and 2% paraformaldehyde in phosphate buffer (pH 7.4) for 1 h at 4 °C and then postfixed with osmium tetroxide for 40 min at 4 °C. The samples were dehydrated in a graded series of ethanol. The samples were treated with a graded propylene oxide series and embedded into Epon. The sections were then sectioned ultra-thin at 80 nm and placed on a copper grid. The final samples were stained with uranyl acetate and lead citrate. The samples were observed using a transmission electron microscope (JEOL-2100F, USA, 200 kV) at the Korea Basic Science Institute, Chuncheon, Republic of Korea. Mitochondrial count, mitochondrial area and cristae area were measured using ImageJ. To calculate cristae volume density, cristae area was divided by the area of the mitochondrion^20^.

### Statistical analysis

All data are represented as the mean ± SEM. Statistically significant differences were assessed by Student’s *t* test and ANOVA. Statistical analyses were performed using Microsoft Excel or GraphPad Prism 9.3.1 (RRID:SCR_002798). All of the significance is expressed as ^*^P < 0.05, ^**^P < 0.01, ^***^P < 0.001, ^$$^*P* < 0.01.

## Results

### An increase in *Thrap3* is associated with NAFLD

**Fig. 1:**
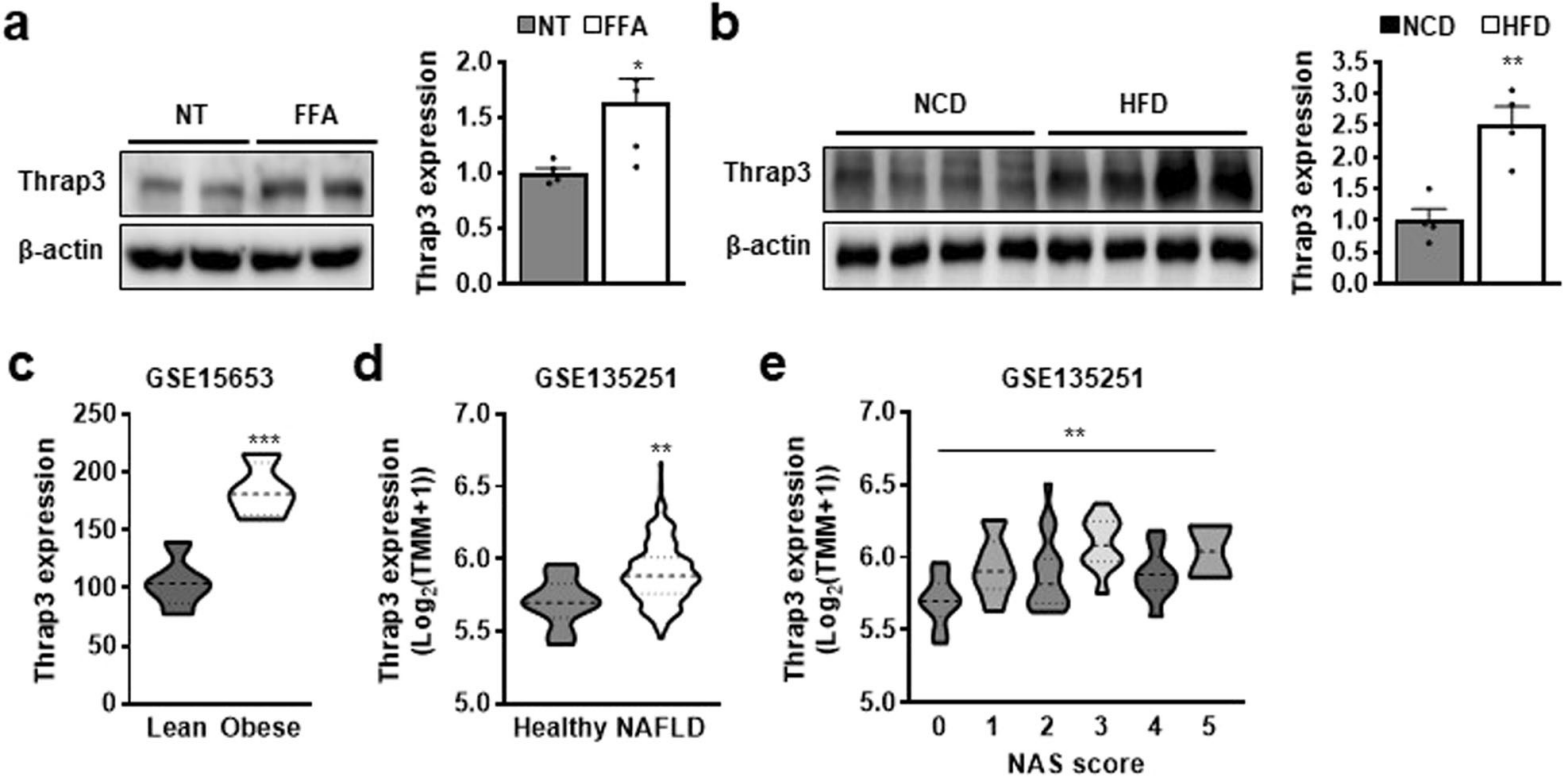
Increased *Thrap3* expression level is associated with NAFLD. **a** The expression of Thrap3 was measured in HepG2 cells treated with FFA for 24 h. The expression level was normalized to no treatment (NT) (*n* = 5 per group). Values represent means ± SEMs. ^*^P < 0.05 vs. NT; **b** Hepatic Thrap3 levels from C57BL/6 J mice fed a high-fat diet (HFD) were normalized to those from C57BL/6 J mice fed a normal chow diet (NCD) (*n* = 4 per group). Values represent means ± SEMs. ^**^P < 0.01 vs. NCD; **c**–**e** Thrap3 expression was analyzed in the livers of obese patients (GSE15653) (Lean, *n* = 5; Obese, *n* = 4) and the livers of NAFLD patients (GSE135251) (**d**, **e**). Thrap3 expression was compared across NAS scores in NAFL patients by one-way ANOVA (**e**). ^***^P < 0.001 vs. Lean (**c**),^**^P < 0.01 (**d**, **e**).

To evaluate the functional role of Thrap3 in NAFLD, we examined Thrap3 expression in various hepatic steatosis-mimicking conditions, including cell lines and mouse models. Human HepG2 cells were stimulated with free fatty acids (FFAs) to establish in vitro hepatocyte steatosis models, and Thrap3 expression was measured. Thrap3 expression was markedly upregulated in FFA-treated HepG2 cells (Fig. 1a). Next, we examined whether Thrap3 was also increased in an in vivo NAFLD mouse model of high-fat diet (HFD)-fed mice. Hepatic Thrap3 expression was upregulated approximately 2.5-fold in HFD-fed mice compared with normal chow diet (NCD)-fed mice (Fig. 1b). Interestingly, this upregulation was also found in an in silico analysis of a public dataset from Gene Expression Omnibus (GEO). We found that *Thrap3* expression was significantly elevated in the livers of obese patients compared with those of lean individuals (GEO accession GSE15653)^13^ (Fig. 1c). Moreover, *Thrap3* expression was upregulated in NAFLD and showed a significant tendency to increase with the increase in the NAFLD activity score (NAS) in the liver of nonalcoholic fatty liver (NAFL) patients (GEO accession GSE135251 and GSE130970)^14–16^ (Fig. 1d, e, Supplementary Fig. 1a). In addition, we observed that Thrap3 expression was positively correlated with histological fatty changes in normal sections from livers of hepatocellular carcinoma patients (Supplementary Fig. 1b, c). Taken together, these results imply a significant correlation between Thrap3 expression and NAFLD, wherein Thrap3 might play a crucial role in the pathogenesis of NAFLD.

### *Thrap3* knockout improved the NAFLD phenotype

**Fig. 2:**
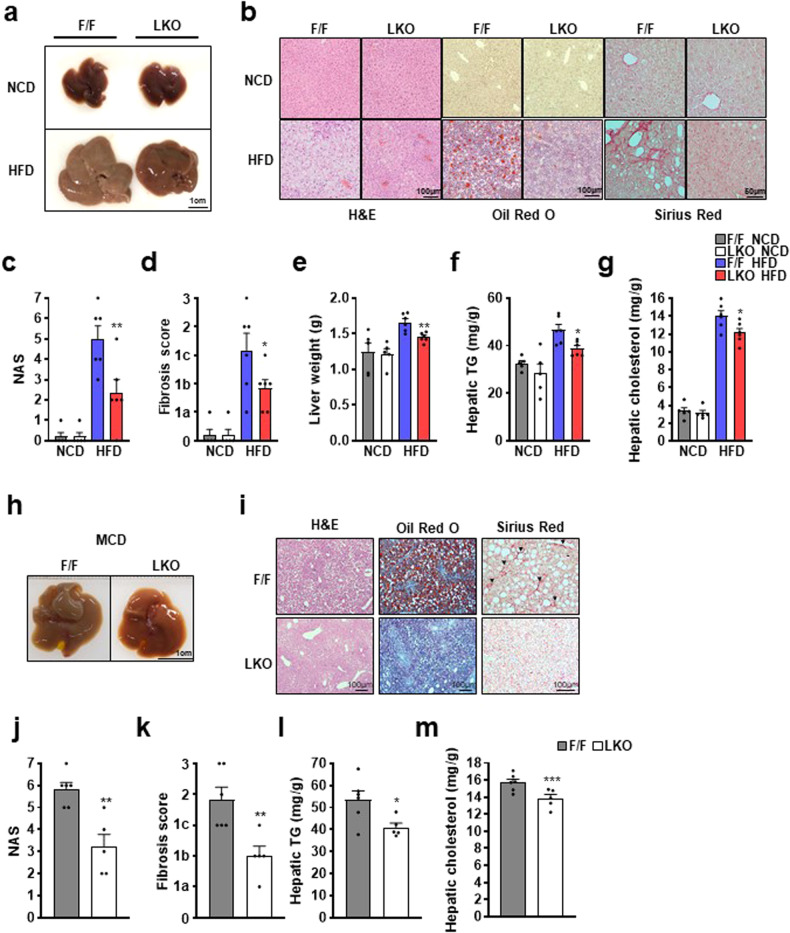
*Thrap3* knockout improved the NAFLD phenotype. **a–g** Thrap3 F/F and Thrap3 LKO mice were fed a normal chow diet (NCD, *n* = 5 per group) or a high-fat diet (HFD, *n* = 6 per group) for 12 weeks. Representative images of liver, H&E staining, Oil Red O staining and Sirius Red staining of liver slides, NAFLD activity score (NAS), fibrosis score, liver weight, hepatic TG, and hepatic cholesterol were analyzed in the indicated mice. Values represent means ± SEMs. ^*^P < 0.05,^**^P < 0.01 vs. F/F HFD; **h–m** Thrap3 F/F (*n* = 6) and Thrap3 LKO (*n* = 5) mice were fed a methionine- and choline-deficient (HCD) diet for 4 weeks. Representative images of liver, H&E staining, Oil Red O staining and Sirius Red staining of liver slides, NAS, fibrosis score, hepatic TG, and hepatic cholesterol were analyzed in the indicated mice. Values represent means ± SEMs. ^*^P < 0.05,^**^P < 0.01,^***^P < 0.001 vs. F/F.

To study hepatic Thrap3 function in vivo, especially in the NAFLD mouse model, we generated liver-specific *Thrap3* knockout mice (referred to as ‘Thrap3 LKO’) by crossing floxed *Thrap3* mice (referred to as ‘wild’ or ‘Thrap3 F/F’) with albumin-Cre mice^21^ (Supplementary Fig. 2a). Quantitative PCR and immunoblotting indicated significant suppression of Thrap3 expression in the liver, while the expression in other organs was not altered (Supplementary Fig. 2b, c), suggesting that Thrap3 expression was mostly from hepatocytes, and liver-specific knockout was well established. To investigate the effects of liver-specific knockout of *Thrap3*, we first analyzed changes in lipid accumulation upon removal of Thrap3 expression in the liver. Wild-type and Thrap3 LKO mice showed no differences in liver size, weight or lipid accumulation when fed a NCD (Fig. 2a–g). However, in the NAFLD mimic HFD model, Thrap3 LKO mice exhibited attenuated HFD-induced hepatic steatosis. While the livers of wild-type mice were enlarged and looked pale, the livers of Thrap3 LKO mice were smaller and darker (Fig. 2a). Liver weight was also reduced in the Thrap3 LKO mice (Fig. 2e). Consistently, the results of H&E, Oil Red O and Sirius Red staining revealed an apparent decrease in lipid accumulation, NAS score and fibrosis in liver sections of Thrap3 LKO mice (Fig. 2b–d). The direct measurement of lipid content in the liver also showed that Thrap3 deficiency led to a decrease in hepatic TGs (Fig. 2f, g). The effects of Thrap3 were also confirmed in methionine/choline-deficient (MCD) diet-induced NAFLD models. The livers of Thrap3 LKO mice were darker in color than those of wild-type mice (Fig. 2h) and showed reduced lipid accumulation, NAS scores and fibrosis on the MCD diet (Fig. 2i–m). Collectively, these data suggest that Thrap3 deficiency reduces the symptoms of NAFLD and that Thrap3 is crucial for the progression of NAFLD.

### Hepatic Thrap3 deficiency improves systemic metabolic homeostasis

**Fig. 3:**
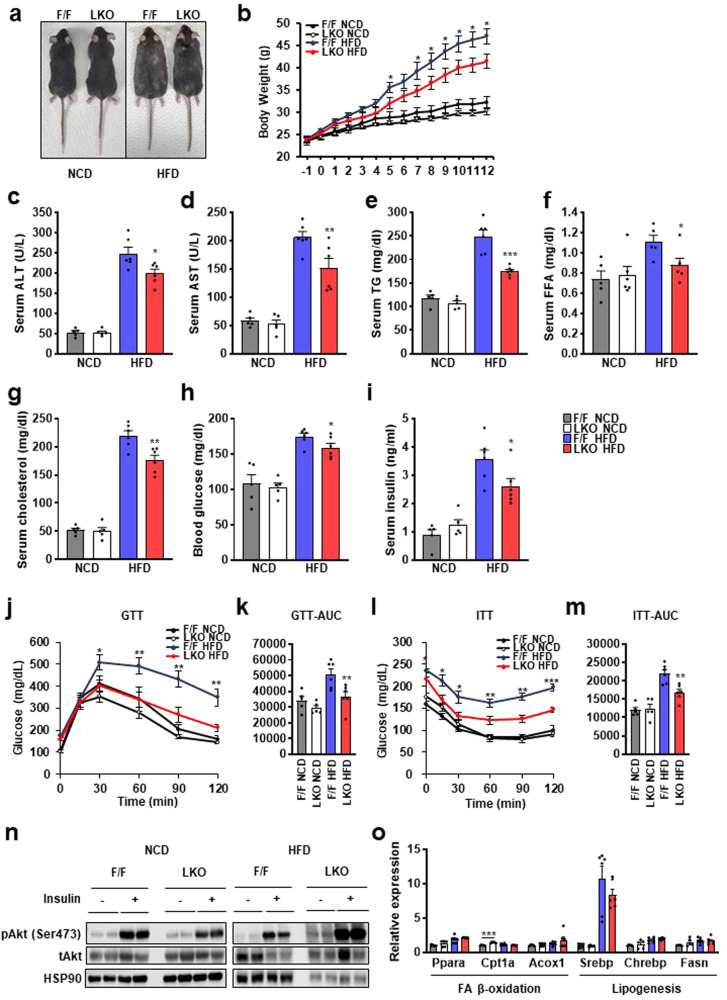
Depletion of *Thrap3* improved metabolic parameters without expression changes in FA β-oxidation and lipogenesis. **a–m** Thrap3 F/F and Thrap3 LKO mice were fed a normal chow diet (NCD, *n* = 5 per group) or a high-fat diet (HFD, *n* = 6 per group) for 12 weeks. Representative liver images of mice, body weight, serum ALT, serum AST, serum TG, serum FFA, serum cholesterol, blood glucose, serum insulin, glucose tolerance (GTT), area under the curve (AUC) of GTT, insulin tolerance (ITT), and AUC of ITT were analyzed in the indicated mice. Values represent means ± SEMs. ^*^P < 0.05, ^**^P < 0.01, ^***^P < 0.001 vs. F/F HFD; **n** Insulin signals were analyzed in the livers of the indicated mice 20 min after insulin (0.75 U/kg) injection; **o** Genes related to FA β-oxidation and lipogenesis were determined by quantitative RT‒PCR. Relative values are normalized to Thrap3 F/F NCD (NCD, *n* = 5 per group; HFD, *n* = 6 per group). Values represent means ± SEMs. ^***^P < 0.001.

Next, we investigated whether Thrap3 deficiency improved systemic metabolic homeostasis. Under NCD conditions, Thrap3 deficiency did not show any significant effects on body weight, metabolic parameters, glucose tolerance or insulin tolerance. However, the bodyweight of Thrap3 LKO mice was significantly lower than that of wild-type mice on an HFD (Fig. 3a, b). Most metabolic parameters, including serum alanine aminotransferase (ALT), serum aspartate aminotransferase (AST), serum TG, FFA, cholesterol, blood glucose and serum insulin, were also improved in HFD-fed Thrap3 LKO mice (Fig. 3c–i). Thrap3 LKO mice also displayed improved glucose tolerance and insulin sensitivity during the glucose tolerance test (GTT) and insulin tolerance test (ITT) (Fig. 3j–m). To clarify whether Thrap3 deficiency could improve insulin-mediated signaling, we performed western blot analysis following insulin administration. The results showed increased phosphorylation of the Ser473 residue of AKT in the insulin-stimulated livers of Thrap3 LKO mice fed both a HFD and NCD (Fig. 3n). Despite the reduced hepatic lipid accumulation and metabolic improvement, including insulin signaling in the liver of Thrap3 LKO mice, hepatic expression of FA uptake, FA β-oxidation, and lipogenesis regulation genes, such as *Ppara* and *Srebp*, were not significantly altered (Fig. 3o, Supplementary Fig. 3). Taken together, these results demonstrate that liver-specific deletion of Thrap3 confers systemic protection against diet-induced obesity and NAFLD without affecting the expression of FA β-oxidation- and lipogenesis-associated genes.

### Thrap3 deficiency promotes autophagosome formation

Autophagy has the crucial functions of maintaining energy homeostasis and removing damaged cellular constituents. Recently, autophagy has been considered important to the development of NAFLD^22^. Interestingly, the beneficial metabolic effects of Thrap3 LKO were similar to those found in the hepatic deletion of autophagy genes. Rubicon is a negative regulator of autophagy, and its deletion ameliorated liver steatosis through enhanced autophagy in HFD-fed mice without altering the expression of lipogenesis- and FA β-oxidation-associated genes^23^. Improved autophagy by acyl-CoA oxidase 1 (Acox1) KO also resulted in similar protection against hepatic steatosis without affecting the expression of genes involved in lipogenesis and FA oxidation^24^. Moreover, mitochondrial β-oxidation showed a corresponding increase with enhanced autophagy in Acox1 KO mice, similar to Thrap3 LKO mice. Conversely, impaired autophagy by ablation of apoptosis signal-regulating kinase 1 (ASK1) accelerated HFD-induced hepatic steatosis with similar expression levels of lipogenesis and FA β-oxidation genes as the wild type^25^. This similarity between Thrap3 LKO and autophagy regulator gene-modified mice suggests a possible relationship between Thrap3 and autophagy regulation.

**Fig. 4:**
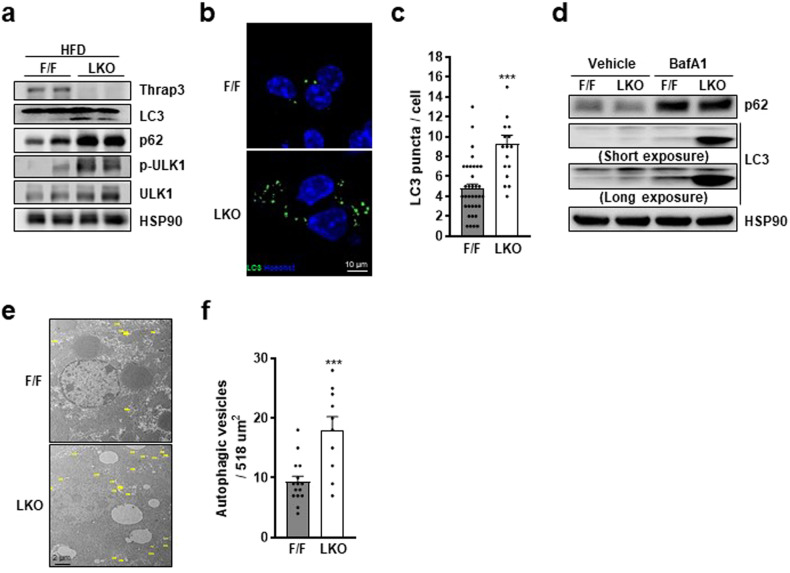
Thrap3 regulated autophagy. **a** Liver lysates from HFD-fed mice were subjected to immunoblot analysis using the indicated antibodies; **b**, **c** Primary hepatocytes were transfected with GFP-LC3, and GFP-LC3 puncta were analyzed using fluorescence microscopy (F/F, *n* = 35; LKO, *n* = 17). Values represent means ± SEMs. ^***^P < 0.001 vs. F/F; **d** Lysates from primary hepatocytes treated with BafA1 (10 nM, 24 h) were subjected to immunoblot analysis using the indicated antibodies; **e**, **f** Livers from HFD-fed mice were analyzed using a transmission electron microscope (EM). Yellow arrows indicate autophagosomes/autolysosomes (**e**). The number of autophagic structures was determined from the EM images (F/F, *n* = 15; LKO, *n* = 10) (**f**). Values represent means ± SEMs. ^***^P < 0.001 vs. F/F.

To determine the effect of Thrap3 deficiency on autophagy, we assessed the expression of autophagosome proteins. Microtubule-associated protein 1A/1B-light chain 3 (LC3) expression was specifically associated with autophagosomes, and Thrap3 LKO mice showed increased hepatic expression of LC3 (Fig. 4a). Similarly, the expression levels of the autophagy-initiating kinase ULK1 and the autophagy receptor p62 were also increased in the livers of Thrap3 LKO mice. To confirm enhanced autophagy, we determined LC3 puncta formation using GFP-LC3. Primary hepatocytes from Thrap3 LKO mice exhibited more LC3-positive structures than those from wild-type mice (Fig. 4b, c). In addition, autophagic flux to autolysosomes was confirmed by using bafilomycin A1 (BafA1), a blocker that prevents the formation of autolysosomes by targeting vacuolar-type H^+^-ATPase^26^. The difference in the amount of LC3 between BafA1-untreated or BafA1-treated hepatocytes was higher in Thrap3 LKO than in wild-type hepatocytes (Fig. 4d). These results reveal that Thrap3 LKO enhances autophagy. The increased occurrence of autophagy was further confirmed in the livers of HFD-fed Thrap3 LKO and wild-type mice by electron microscopy (EM) (Fig. 4e, f).

### Thrap3 deficiency increases energy expenditure through mitochondrial quality control

**Fig. 5:**
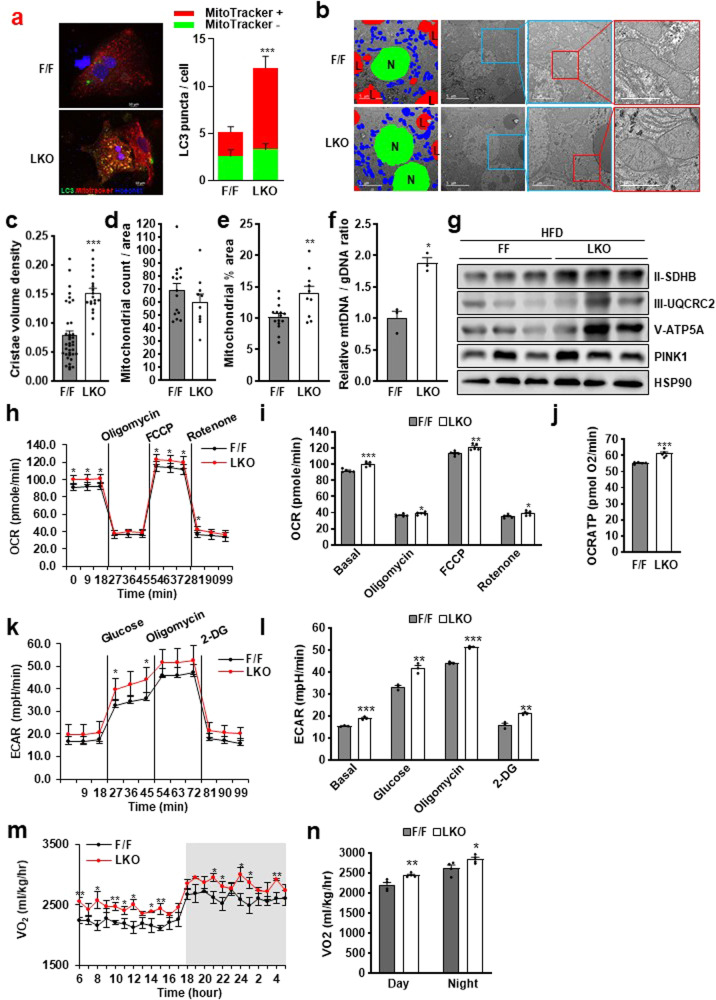
Thrap3 regulated energy expenditure through mitochondrial quality control. **a** Primary hepatocytes were transfected with GFP-LC3 and stained with MitoTracker. GFP-LC3 puncta and MitoTracker were analyzed using fluorescence microscopy (F/F, *n* = 11; LKO, *n* = 10). Values represent means ± SEMs. ^***^P < 0.001 vs. F/F; Mitochondria of liver from HFD-fed mice were analyzed using a transmission electron microscope (EM). Representative electron micrographs reveal nuclei (green; N), lipid droplets (red; L) and mitochondria (blue) (**b**). Cristae volume density (F/F, *n* = 37; LKO, *n* = 20) (**c**), number of mitochondria (**d**) and % area (**e**) were determined from the EM images (F/F, *n* = 15; LKO, *n* = 10). Values represent means ± SEMs.^***^P < 0.001, ^**^P < 0.01 vs. F/F; **f** The mitochondrial DNA copy number was determined by the ratio of the mitochondrial DNA gene *Polg* to the nuclear DNA gene *Actb* (*n* = 3 per group). Values represent means ± SEMs. ^*^P < 0.05 vs. F/F; **g** The mitochondrial respiratory chain complex proteins and mitophagy markers were determined in HFD-fed mice; **h–l** Oxygen consumption rate (OCR) (**h, i**) (*n* = 5 per group), OCRATP levels (**j**) (*n* = 5 per group) and extracellular acidification rate (ECAR) (**k, l**) (*n* = 3 per group) were measured in primary hepatocytes from the indicated mice. Values represent means ± SEMs. ^*^P < 0.05, ^**^P < 0.01, ^***^P < 0.001 vs. F/F; **m, n** The consumption rate (VO_2_) of HFD-fed mice was measured using CLAMS (*n* = 4 per group). Values represent means ± SEMs. ^*^P < 0.05, **P < 0.01 vs. F/F.

In NAFLD patients, mitochondria are damaged and have a wide range of functional and structural abnormalities, and autophagy maintains mitochondrial quality by removing damaged mitochondria (mitophagy)^27^. In this context, we assumed that increased autophagy in Thrap3 LKO mice contributed to the metabolic improvement shown in Thrap3 LKO mice through mitochondrial quality control. To substantiate our hypothesis, we determined the colocalization of LC3 puncta and MitoTracker, a mitochondrial-specific dye. Most of the increased LC3 puncta in FFA-treated Thrap3 LKO hepatocytes were colocalized with MitoTracker (Fig. 5a). This finding indicates that deletion of Thrap3 facilitates mitophagy.

HFD disintegrates the cristae structure and induces mitochondrial damage, which decreases the mitochondrial DNA (mtDNA) level and OXPHOS capacity^28^. Damaged mitochondria are removed by autophagy to maintain mitochondrial number and functional quality^29^. As shown in Fig. 5b, disruption of autophagy due to a HFD resulted in the accumulation of dysfunctional mitochondria in wild-type mice. In contrast, HFD-fed Thrap3 LKO mice had more mitochondria with clear cristae structures and increased volume density in the liver (Fig. 5b, c). Although the livers of Thrap3 LKO mice exhibited an insignificant decrease in mitochondrial number (*P* = 0.29), the area of mitochondria and mtDNA levels were increased compared with those of wild-type mice (Fig. 5d–f). Accompanying elevated PINK1, a marker protein of mitophagy, mitochondrial respiratory chain complex proteins, such as succinate dehydrogenase complex iron sulfur subunit B (SDHB), cytochrome b-c1 complex subunit 2 (UQCR2), and ATP synthase subunit alpha (ATP5A), were also increased in the livers of Thrap3 LKO mice compared with wild-type mice (Fig. 5g). Mitochondrial function analyzed *via* the oxygen consumption rate (OCR) was also improved in hepatocytes from Thrap3 LKO mice (Fig. 5h, i). Furthermore, ATP production and the extracellular acidification rate (ECAR) were increased in hepatocytes from Thrap3 LKO mice compared with those from wild-type mice (Fig. 5j–l). Consistent with these results, Thrap3 LKO mice showed an increased oxygen consumption rate without changes in locomotor activity or food intake under HFD conditions (Fig. 5m, n). Taken together, these results suggest that Thrap3 deficiency improves energy expenditure through enhanced mitochondrial function by regulating autophagy in HFD-fed mice.

### Thrap3 regulates autophagy through AMPK

**Fig. 6:**
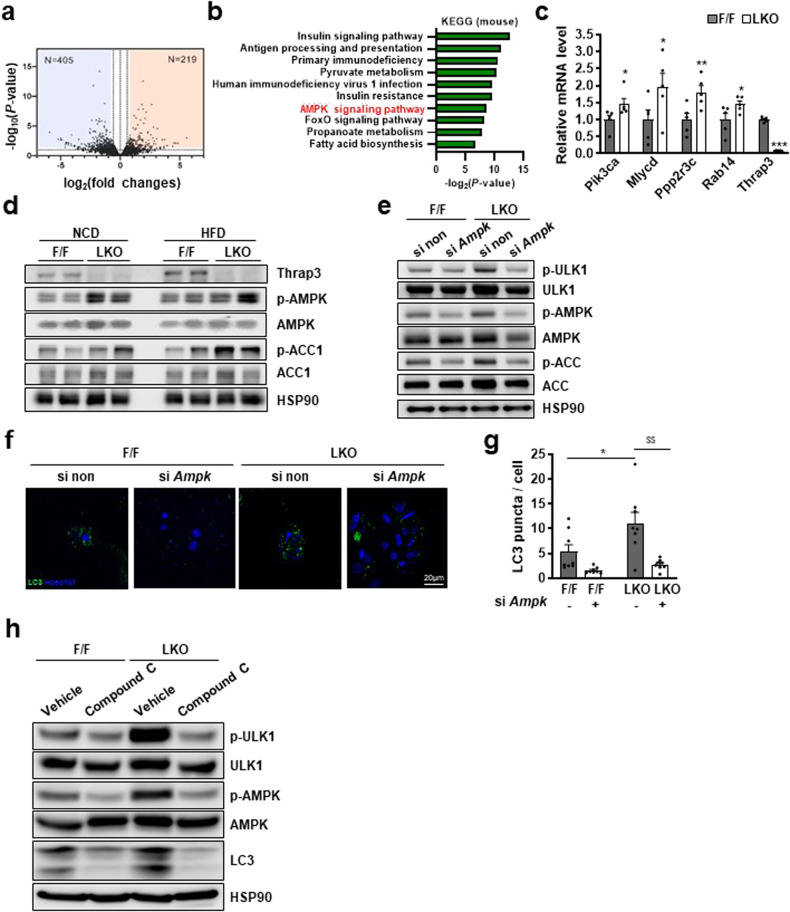
Thrap3 regulated autophagy through AMPK. **a** RNA-seq was performed on samples from the livers of NCD-fed Thrap3 F/F and Thrap3 LKO mice. Volcano plot of the gene expression of Thrap3 LKO (log_2_-fold change) compared to that of Thrap3 F/F from RNA-seq analysis; **b** Gene ontology enrichment analysis of differentially expressed genes in Thrap3 LKO compared to Thrap3 F/F; **c** Genes related to the AMPK signaling pathway were identified in the livers of HFD-fed Thrap3 F/F and Thrap3 LKO mice by quantitative RT‒PCR (*n* = 5 per group). Relative values are normalized to Thrap3 F/F. Values represent means ± SEMs. ^*^P < 0.05, ^**^P < 0.01, ^***^P < 0.001 vs. F/F; **d** Western blot analysis of phospho-AMPK, AMPK, phospho-ACC and ACC levels in the livers of NCD- or HFD-fed mice; **e** Western blot analysis of phospho-ULK1, ULK1, phospho-AMPK, AMPK, phospho-ACC and ACC levels in primary hepatocytes from indicated mice with nontargeting siRNA (si non) or *Ampk*-targeting siRNA (si *Ampk*); **f,** **g** Primary hepatocytes were transfected with GFP-LC3 and nontargeting siRNA (si non) or *Ampk*-targeting siRNA (si *Ampk)*. GFP-LC3 puncta were analyzed using confocal microscopy (*n* = 8 per group). Values represent means ± SEMs. ^*^P < 0.05 vs. F/F si non. ^$$^*P* < 0.01 vs. LKO si non; **h** Western blot analysis of phospho-ULK1, ULK1, phospho-AMPK, AMPK, and LC3 levels in primary hepatocytes from indicated mice incubated with vehicle or 10 μM Compound C for 24 h.

To determine the mechanism underlying the causal relationship between Thrap3 deficiency and metabolic improvement through enhanced autophagy, we performed RNA-seq and profiled the liver transcriptomes of wild-type and Thrap3 LKO mice. The gene expression from Thrap3 LKO mice showed a distinct expression pattern compared with wild-type mice, implying a substantial impact of Thrap3 on metabolic gene expression. Thrap3 deficiency significantly (*P* < 0.01, 2-fold change) upregulated the expression of 219 genes and downregulated the expression of 405 genes (Fig. 6a). The gene ontology enrichment analysis of differentially expressed genes (DEGs) revealed that Thrap3 deficiency activated genes in pathways related to the ‘insulin signaling pathway’ and ‘pyruvate metabolism’ (Fig. 6b), in agreement with the enhanced insulin signaling observed in Thrap3 LKO mice (Fig. 3n). Interestingly, bioinformatic analysis also showed that genes related to the ‘AMPK signaling pathway’ were activated in Thrap3 LKO mice, and accordingly, we corroborated the increased expression of AMPK signaling genes in HFD-fed Trap3 LKO mice (Fig. 6c). The phosphorylation of AMPK and its target ACC was also increased in Thrap3 LKO mice without any change in their expression under both NCD- and HFD-fed conditions (Fig. 6d).

AMPK is a major sensor of cellular energy status, and an agonist of AMPK counteracts NAFLD by increasing autophagy^8^. We hypothesized that the metabolic improvement effect of Thrap3 deficiency was mediated by AMPK. To test this hypothesis, we determined the effect of AMPK knockdown on Thrap3 knockout mice. Primary hepatocytes from Thrap3 LKO mice showed improved autophagy with increased AMPK activation (Fig. 6e–g). AMPK knockdown attenuated ULK1 phosphorylation, which was increased in Thrap3 LKO mice (Fig. 6e). The increase in LC3-positive structures due to Thrap3 deficiency was significantly reduced by AMPK knockdown (Fig. 6f, g). To clarify the role of AMPK, we tested the effect of Compound C, an AMPK inhibitor, on LC3 expression. AMPK inhibition reduced the increased phosphorylation of ULK1 and expression of LC3 in primary hepatocytes from Thrap3 LKO mice (Fig. 6h). These results suggest that autophagy regulation by Thrap3 is mediated by AMPK.

### Thrap3 regulated cytosolic translocation of AMPK via direct interaction

**Fig. 7:**
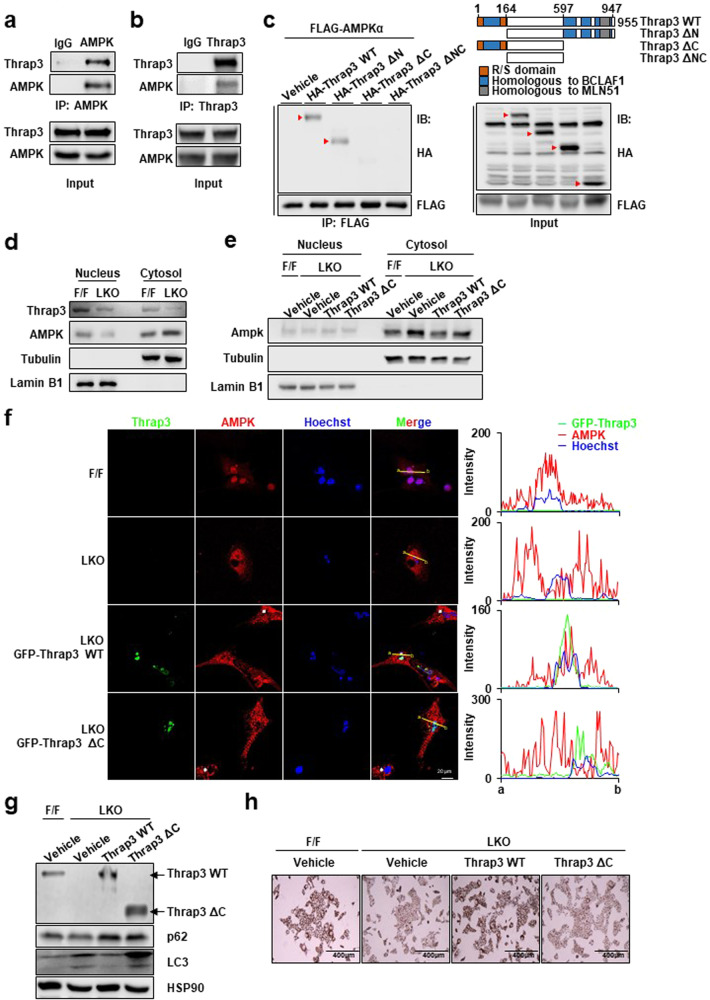
Thrap3 regulated cytosolic translocation of AMPK via direct interaction. **a,** **b** HepG2 cell lysates were immunoprecipitated with normal IgG (IgG), an anti-AMPK antibody or an anti-Thrap3 antibody. Precipitates and cell lysates were subjected to immunoblotting for Thrap3 and AMPK; **c** Schematic of the Thrap3 mutant with functional domain deletion. HepG2 cells were transfected with FLAG-tagged AMPKα and HA-tagged Thrap3 mutant. The cells were then immunoprecipitated with an anti-FLAG antibody. Precipitates and cell lysates were subjected to immunoblotting for FLAG and HA; **d** Nucleus and cytosolic fractions were isolated from the indicated primary hepatocytes and subjected to immunoblotting for Thrap3, AMPK, Tubulin and Lamin B1; **e** Nucleus and cytosolic fractions were isolated from the indicated DNA-transfected primary hepatocytes and subjected to immunoblotting for AMPK, Tubulin and Lamin B1; **f** Primary hepatocytes from Thrap3 F/F and Thrap3 LKO mice were transfected with GFP-Thrap3 WT or GFP-Thrap3 ΔC and immunostained with anti-AMPK antibody. Representative confocal images reveal GFP-Thrap3 WT or GFP-Thrap3 ΔC (green), AMPK (red) and nuclear DNA (blue). The white arrow indicates untransfected Thrap3 LKO hepatocytes. Fluorescence intensity profiles of GFP-Thrap3 WT or GFP-Thrap3 ΔC (green), AMPK (red) and nuclear DNA (blue) signals across nuclei (from a to b) were measured using confocal microscopy; **g** Lysates from the indicated DNA-transfected primary hepatocytes were subjected to immunoblotting for the indicated proteins; **h** Representative images of Oil Red O staining from the indicated DNA-transfected primary hepatocytes incubated with FFA for 24 h.

Thrap3 regulates DNA damage, RNA processing and transcription. Moreover, Thrap3 functions by binding with DDX5, eIF4AIII and PPARγ^9,30,31^. In the context of the interaction-mediated function, we tested whether Thrap3 regulates AMPK activation by binding to it. By performing an immunoprecipitation assay, we found that Thrap3 directly interacted with AMPK. By using an anti-AMPK antibody in hepatocytes, we determined that Thrap3 coimmunoprecipitated with AMPK (Fig. 7a). Conversely, AMPK was also precipitated by an anti-Thrap3 antibody (Fig. 7b). To further investigate the binding between Thrap3 and AMPK, recombinant Thrap3 fragments were constructed as HA-tagged N-terminus-deleted (ΔN), C-terminus-deleted (ΔC), or both terminus-deleted Thrap3 (ΔNC). The results showed that HA-Thrap3 WT and HA-Thrap3 ΔN bound AMPK. However, the interaction between Thrap3 and AMPK was abrogated in both the ΔC and ΔNC constructs of the HA-Thrap3 protein (Fig. 7c). These results imply that Thrap3 negatively regulates AMPK through direct interaction at its C-terminal region.

Thrap3 mainly exists in the nucleus^32^. Interestingly, AMPK shuttles between the nucleus and cytoplasm, and AMPK function is regulated by this translocation, which is tightly regulated by multiple factors, such as starvation and heat shock^33,34^. To determine whether Thrap3 influences the translocation of AMPK, we isolated the nuclear and cytosolic fractions from FFA-treated primary hepatocytes of wild-type and Thrap3 LKO mice. AMPK was significantly decreased in the nuclear fraction and increased in the cytosolic fraction in hepatocytes of Thrap3 LKO mice (Fig. 7d). To investigate the effect of Thrap3 on AMPK translocation, we overexpressed Thrap3 WT or Thrap3 ΔC in Thrap3 LKO hepatocytes. Nuclear AMPK was increased by overexpression of Thrap3 WT in FFA-treated Thrap3 LKO hepatocytes, while it was not increased by Thrap3 ΔC (Fig. 7e). Increased cytosolic AMPK was also significantly reduced by Thrap3 WT in Thrap3 LKO hepatocytes but not by Thrap3 ΔC. For further validation, we tested the translocation of AMPK and colocalization with Thrap3 in the nucleus using immunofluorescence with GFP-Thrap3 expression in FFA-treated Thrap3 LKO hepatocytes. Consistent with previous results, Thrap3 LKO hepatocytes showed reduced AMPK in the nucleus (Fig. 7f, white arrow), and GFP-Thrap3 WT trapped AMPK and colocalized in the nucleus, but GFP-Thrap3 ΔC did not interact with AMPK (Fig. 7f).

To determine whether the interaction between the Thrap3 C-terminus and AMPK is linked to the phenotypes of Thrap3 LKO, we analyzed the effect of Thrap3 WT and ΔC on autophagy and lipid accumulation. As shown in Fig. 7g, ectopic expression of Thrap3 WT reduced the expression of LC3 in hepatocytes from Thrap3 LKO mice; however, Thrap3 ΔC could not reduce the increased LC3 expression of hepatocytes from Thrap3 LKO mice. In addition, Thrap3 ΔC could not restore lipid accumulation in hepatocytes from Thrap3 LKO mice, although ectopic expression of Thrap3 WT increased lipid accumulation (Fig. 7h). These results strongly indicate that binding of the Thrap3 C-terminus to AMPK is important for Thrap3-mediated regulation of autophagy and lipid accumulation, reflecting the phenotype of Thrpa3 LKO mice.

Taken together, these results suggest that Thrap3 inhibits AMPK activity by regulating AMPK translocation through its C-terminus, which further inhibits AMPK-mediated autophagy and mitochondrial quality control and consequently exacerbates NAFLD.

## Discussion

**Fig. 8:**
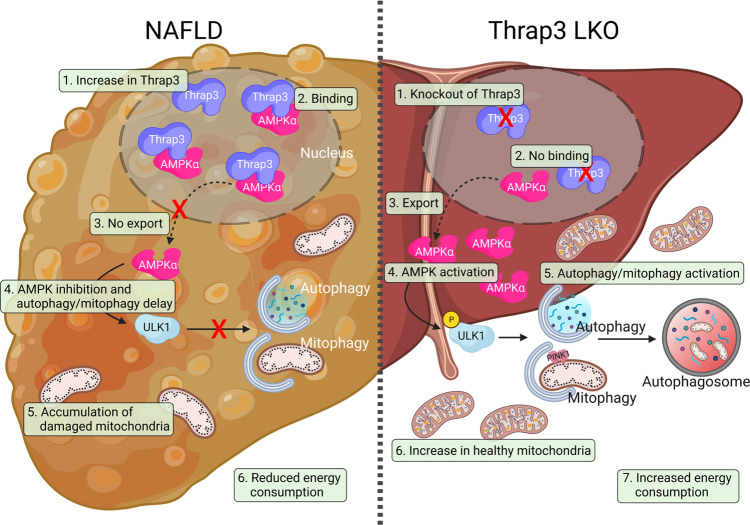
Schematic diagram of the mechanism by which Thrap3 affects NAFLD through translocation of AMPK. Dysregulation of autophagy/mitophagy contributes to the progression of nonalcoholic fatty liver disease (NAFLD). In NAFLD, increased Thrap3 suppresses autophagy/mitophagy by sequestering AMPK in the nucleus. Inhibition of mitophagy does not effectively remove damaged mitochondria and maintain healthy mitochondria, and as a result, it leads to a decrease in energy consumption and exacerbates NAFLD progression. Liver-specific Thrap3 knockout mice show improved lipid accumulation, metabolic parameters, and mitochondrial function and enhanced autophagy/mitophagy in the NAFLD model. Thrap3 may be a potential therapeutic target for preventing and treating NAFLD by regulating the AMPK/autophagy/mitophagy axis.

Thrap3 has been reported as an RNA-binding protein or transcription cofactor that regulates alternative splicing of pre-mRNAs, cancer cell growth, differentiation and DNA damage^9,30,35^. However, the function of Thrap3 in energy metabolism remains largely unknown. In a previous study, we showed that Thrap3 controls diabetic gene programming by binding to the phosphorylated Ser273 residue of PPARγ in adipose tissues^9^. Here, we found that hepatic Thrap3 impaired autophagy and mitochondrial function in NAFLD by altering the cytosolic translocation of AMPK. In contrast to our previous study showing that Thrap3 regulates metabolic processes through PPARγ, hepatic deletion of Thrap3 improved autophagy without altering the expression of genes involved in lipogenesis and FA β-oxidation. Whether interaction with PPARγ is also involved in Thrap3-mediated regulation of autophagy will be addressed in follow-up studies, although the role of PPARγ in autophagy remains controversial^36,37^. Taken together, our results imply that Thrap3 plays a crucial role in metabolic regulation through the suppression of the AMPK-autophagy axis (Fig. 8). Further studies should focus on the role of Thrap3 in other metabolic diseases and in understanding the underlying mechanisms.

In response to cellular energy status, mitochondrial content and mass are exquisitely regulated through mitochondrial turnover and the maintenance of a healthy and active mitochondrial pool^38^. In the early phase of HFD feeding, an increase in the substrate for OXPHOS induces an increase in mitochondria and mtDNA as a compensatory mechanism. However, mitochondrial morphology and synthesis of mitochondrial machinery remain unaltered, and respiratory chain proteins are distributed among the greater number of mitochondria. These changes impair mitochondrial oxidative capacity and antioxidant defense, increasing damaged mitochondria, although total mitochondria mass is increased^39^. Here, we showed that Thrap3 LKO increased energy expenditure and healthy mitochondrial content concomitant with increased mitophagy with elevated PINK1 expression (Fig. 5). PINK1/Parkin-mediated mitophagy functions in mitochondrial quality control. In response to mitochondrial damage, such as membrane depolarization and complex dysfunction, PINK/Parkin accumulates in the outer mitochondrial membrane and induces mitophagy to remove damaged mitochondria^40^. On the other hand, PINK1/Parkin also induces mitochondrial biogenesis, mitochondrial protein synthesis, and import into mitochondria through phosphorylation and ubiquitination of other mitochondrial and cytosolic targets, such as PARIS, hnRNP-F and TOM complex proteins^41–43^. In particular, AMPK, activated in Thrap3 LKO cells, could stimulate both mitophagy and mitochondrial biogenesis^38^. AMPK is also involved in PINK1/Parkin-mediated mitophagy^44^. In our study, Thrap3 LKO increased mitophagy together with mitochondrial area and mtDNA. Although the contribution of mitophagy to the increase in mitochondrial mass was not clearly explored, activated AMPK and PINK1/Parkin pathways could adequately explain the phenotypes of Thrap3 LKO. Moreover, similar to our results, there is a report that increased mitochondrial degradation accompanies an increased rate of mitochondrial activity under high OXPHOS activity conditions^45^.

In response to nutrients, AMPK is activated by an increased cellular ratio of AMP:ATP and ADP:ATP and phosphorylation of the activating site (Thr172) by LKB1, calcium/calmodulin-dependent protein kinase kinase 2 (CAMKK2) and TGF-beta-activated kinase 1 (TAK1)^46,47^. Localization of AMPK is also an important mechanism for regulating the available AMPK pool for certain substrates and the proper functioning of AMPK. AMPK shuttles between the nucleus and cytoplasm to regulate its targets in both compartments^33^. The translocation of AMPK to the nucleus activates transcription factors/cofactors, such as p300/cAMP-regulated enhancer-binding protein (CREB)-binding protein (p300/CBP) and FOXOs, which are regulated by phosphorylation and myristoylation of the β subunit of AMPK^48^. The nuclear localization signal in the α-subunit also influences the localization of AMPK^49^. Restricting AMPK to the nucleus can disturb cytosolic AMPK function. Recently, PARylation of AMPK has been suggested as an autophagy regulatory mechanism that enhances AMPK nuclear export^50^. Suppression of PARylation inhibits the activation of the cytosolic AMPK pool and autophagosome formation. Similarly, Thrap3 sequesters AMPK in the nucleus and suppresses autophagy, thus controlling mitochondrial health in NAFLD conditions.

Autophagy is associated with pathological changes in NAFLD and is decreased in patients with NAFLD^51^. Various mechanisms have been suggested to explain the inhibition of autophagy in fatty liver, including decreased autophagy gene expression and impaired fusion between autophagosomes and lysosomes^52,53^. Reduced hepatic autophagy results in a decreased lipolytic breakdown of TGs and cholesterols from lipid droplets (lipophagy) and consequently a reduction in mitochondrial FA β-oxidation, insulin resistance and increased glucose production^22^. Based on the importance of autophagy, it is considered a promising target for the treatment of NAFLD, and autophagy-targeting agents are being developed.

Autophagy also maintains healthy mitochondria by selectively removing depolarized and damaged mitochondria for metabolic homeostasis^4^. Damaged mitochondria and impaired elimination of damaged mitochondria, known as mitophagy, have been considered etiological factors of NAFLD. Lipotoxicity, triggered by excessive lipid supply, causes mitochondrial dysfunction^54^. Reduced mitophagy contributes to the development and progression of NAFLD by consequently decreasing mitochondrial mass and integrity^29^. Anomalous mitochondria with loss of cristae and reduced activity of respiratory chain enzyme complexes have been described for the first time in NASH patients^55,56^. Reduced capacity for OXPHOS is accompanied by enhanced ROS formation, inducing mitophagy arrest, mtDNA damage and degradation, and further ROS production. This vicious cycle results in the propagation of cell damage^57,58^. Overproduction of ROS aggravates lipid accumulation and activates the NLR family pyrin domain containing 3 (NLRP3) inflammasome, resulting in exacerbation of hepatic steatosis^59^. Therefore, mitophagy serves to prevent this vicious cycle by eliminating dysfunctional mitochondria. In addition, as mentioned previously, mitophagy also contributes to new healthy mitochondria generation through crosstalk with the mitochondrial biogenesis pathway, including mitochondrial protein synthesis and import into the mitochondria^60–62^. Indeed, genetic or pharmacological modulation of mitophagy-associated proteins, such as PINK1, Parkin, acyl-CoA:lysocardiolipin acyltransferase-1 (ALCAT1), and Bnip3, alleviates or exacerbates the NAFLD phenotype through mitophagy regulation^29^. Therefore, the regulation of mitophagy has attracted attention as a new therapeutic strategy for NAFLD treatment^63^. Thrap3 LKO mice exhibited enhanced mitophagy, as evidenced by increased mitophagosomes and PINK1 expression, concurrent with improved mitochondrial functional integrity, as evidenced by increased cristae density, OXPHOS capacity, mtDNA copy number, and respiratory chain protein level (Fig. 5). These results imply that Thrap3 LKO restored mitochondrial dysfunction, prevented the vicious cycle leading to ROS overproduction and hepatic steatosis, and finally ameliorated the NAFLD phenotype. Thus, our results strongly support that the Thrap3-AMPK-autophagy/mitophagy axis could be a potent therapeutic target to combat NAFLD.

## Supplementary information

Supplementary Information

Supplementary Dataset 1

## Data Availability

Data of the differentially expressed genes from RNA-seq are provided in Supplementary Dataset 1. Any additional information required to reanalyze the data reported in this study is available from the lead contact upon request
