# Supplementary material for: Thrap3 promotes nonalcoholic fatty liver disease by suppressing AMPK-mediated autophagy
Source: Exp Mol Med. 2023 Aug 1;55(8):1720–33. doi: 10.1038/s12276-023-01047-4 (PMC10474030; doi:10.1038/s12276-023-01047-4)
Supplement: Supplementary file 1 — Supplementary Information [file 12276_2023_1047_MOESM1_ESM.pdf]

Supplementary Information

Thrap3 promotes nonalcoholic fatty liver disease by suppressing AMPK-mediated autophagy

Hyun-Jun Jang[1,2,*], Yo Han Lee[1,*], Tam Dao[4], Yunju Jo[4], Keon Woo Khim[1], Hye-jin Eom[1], Ju Eun Lee[1], Yi Jin Song[1], Sun Sil Choi[1], Kieun Park[1], Haneul ji[1], Young Chan Chae[1], Kyungjae Myung[5], Hongtae Kim[1], Dongryeol Ryu[4], Neung Hwa Park[3,†], Sung Ho Park[1,†], Jang Hyun Choi[1,†]

[1]Department of Biological Sciences, Ulsan National Institute of Science and Technology (UNIST), Ulsan 44919, Republic of Korea

[2]Herbal Medicine Resources Research Center, Korea Institute of Oriental Medicine, Naju 58245, Republic of Korea

[3]Department of Internal Medicine, University of Ulsan College of Medicine, Ulsan University Hospital, Ulsan 44033, Republic of Korea

[4]Department of Molecular Cell Biology, Sungkyunkwan University (SKKU) School of Medicine, Suwon 16419, Republic of Korea

[5]Center for Genomic Integrity, Institute for Basic Science, Ulsan 44919, Republic of Korea

[†]Corresponding Author:

Jang Hyun Choi, Department of Biological Sciences, Ulsan National Institute of Science and Technology (UNIST), Ulsan 44919, Republic of Korea. Tel.: +82 52 217 2543; Fax: +82 52 217 3219;
E-mail: janghchoi@unist.ac.kr

Sung Ho Park, Department of Biological Sciences, Ulsan National Institute of Science and Technology (UNIST), Ulsan 44919, Republic of Korea. Tel.: +82 52 217 5203
E-mail: parksu@unist.ac.kr

Neung Hwa Park, Department of Internal Medicine, University of Ulsan College of Medicine, Ulsan University Hospital, Ulsan 44033, Republic of Korea.
E-mail: nhparkmd@gmail.com

***These authors contributed equally to this work**

Supplementary Table 1: siRNA sequences.

Name	Sequence
si *Ampk*	AUGAUGUCAGAUGGUGAAUUU

Supplementary Table 2: Primer sequences.

	**Sequences of the primer for qPCR**	
**Gene symbol**	**Forward primer**	**Reverse primer**
Thrap3	TCTCGGTCTCGTTCGTTTTCA	TCCTTTCTCTGTTATGAGCTGGA
Ppara	TCAGGGTACCACTACGGAGT	CTTGGCATTCTTCCAAAGCG
Cpt1a	AGTTCCATGACCCATCTCTGTC	TTCTTCTTCCAGAGTGCAGC
Acox1	TAACTTCCTCACTCGAAGCCA	AGTTCCATGACCCATCTCTGTC
Srebf1	GGAGCCATGGATTGCACATT	CTTCCAGAGAGGAGGCCAG
Chrebp	CATCTCCAGCCTCGTCTTC	CTTGGTCTTAGGGTCTTCAGG
Fasn	GGAGGTGGTGATAGCCGGTAT	TGGGTAATCCATAGAGCCCAG
Gpat1	CTTGGCCGATGTAAACACACC	CTTCCGGCTCATAAGGCTCTC
Gpat2	AGCAGAGGAGTAACCACAATGG	GGGCGATACTTTCCCAGGA
Agpat1	TAAGATGGCCTTCTACAACGGC	GGAACTCTGGTGGTTGGACAC
Agpat2	CAGCCAGGTTCTACGCCAAG	TGATGCTCATGTTATCCACGGT
Dgat1	GTGCCATCGTCTGCAAGATTC	GTGCCATCGTCTGCAAGATTC
Dgat2	GCGCTACTTCCGAGACTACTT	GGGCCTTATGCCAGGAAACT
Cd36	GCGACATGATTAATGGCACAG	GATCCGAACACAGCGTAGATAG
Fatp1	CTGGGACTTCCGTGGACCT	TCTTGCAGACGATACGCAGAA
Fatp2	CGAGACGAGACGCTCACCTA	ACGAATGTTGTAGTTGAGGCAC
Fatp3	ATGGGGCTCCACCTATGGG	TGGCACTGTAGAACCTTCAGA
Fatp4	ACTGTTCTCCAAGCTAGTGCT	GATGAAGACCCGGATGAAACG
Fatp5	TCTATGGCCTAAAGTTCAGGCG	CTTGCCGCTCTAAAGCATCC
Fatp6	GTCATGGCTCACAGGATTGGG	CACGTCCTCGTAGGTGTAGAC
Pik3ca	ACGACCATCTTCGGGTGAAC	CTTCACGGTTGCCTACTGGT
Mlycd	TGCCAAGAAATCTCAGCGGT	TTCTTGGAGCCCAGGTAGGA
Ppp2r3c	TGATGTTGCTGGCCAAGGAT	CAGGGCAGAAGGAGCAGAAA
Rab14	TTCAGAGCGGTTACACGGAG	ACATTCTCTCCCGTTTTTGCG
Tbp	ACCCTTCACCAATGACTCCTATG	TGACTGCAGCAAATCGCTTGG
Polg	TGTGAATATCCCTGGTTGCTG	CTTGGGCAGGAAATCTTTGG
Actb	GGCTGTATTCCCCTCCATCG	CCAGTTGGTAACAATGCCATGT

Supplementary Fig. 1: Hepatic Thrap3 expression is increased with fatty change.

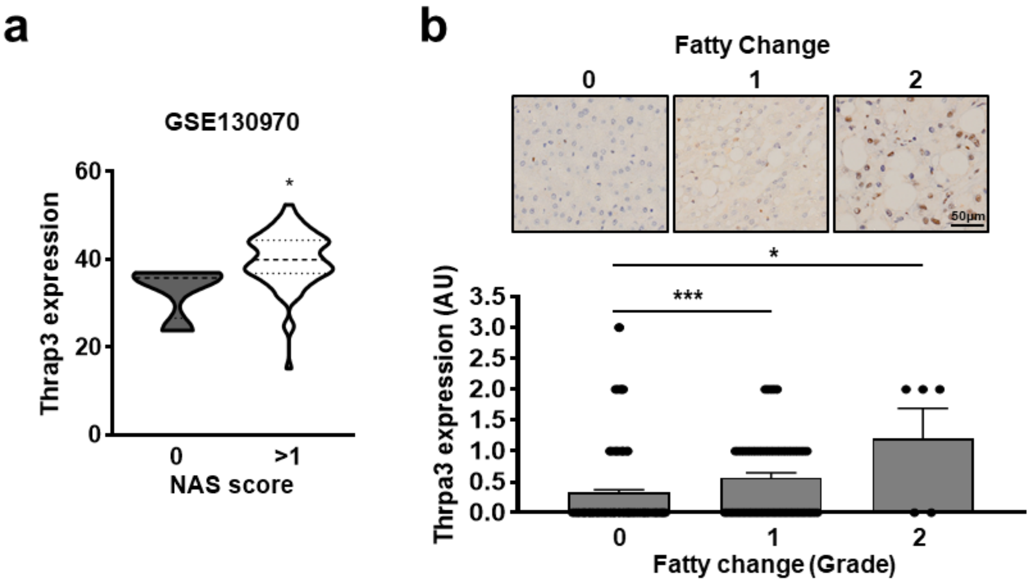

c

Characteristics	Fatty change		
	0	1	2
Age (years)	56.21±10.27	57.79±9.27	58.00±9.92
Weight (kg)	66.59±10.32	68.06±9.59	67.80±19.46
Height (cm)	165.4±7.57	164.5±7.79	161.6±11.95
BMI (kg/m^2)	24.29±2.94	25.09±2.74	25.36±4.25
Male /Female	169/28	51/11	4/2

Mean±SD

Supplementary Fig. 1: Hepatic Thrap3 expression is increased with fatty change.

a *Thrap3* expression was analyzed in the livers of NAFLD patients (GSE130970). *Thrap3* expressions were compared across the NAS score (0, n = 4; >1, n = 74). **P* < 0.05 vs NAS score 0; **b**, **c** Liver sections were stratified into three main groups based on their histological fatty change profiles. The first group (grade 0) represented the control group, which included patients without liver steatosis (n = 197). The second group (grade 1) was represented by patients (n = 62) with mild fatty changes, defined by 5 % to 10 % of hepatocytes exhibiting cytoplasmic lipid droplets. The third group (grade 2) consisted of patients (n = 6) with severe fatty changes, defined by a proportion of hepatocytes containing lipid droplets exceeding 10 %. Expression of Thrap3 was determined with anti-Thrap3 antibody on the normal liver slide of HCC patients stratified into three main groups based on their histological fatty change profiles. Values represent means ± SEM. **P* < 0.05, ****P* < 0.001 vs Fatty change Grade 0 (**b**). Characteristics of patients. Values represent means ± SD (**c**).

Supplementary Fig. 2: Generation of liver-specific *Thrap3* knockout.

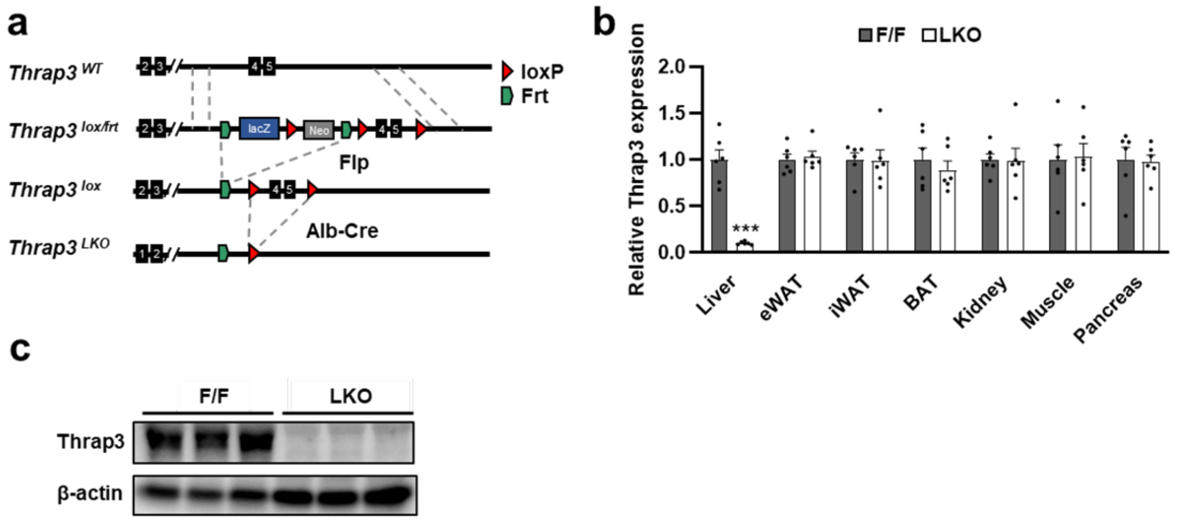

Supplementary Fig. 2: Generation of liver-specific *Thrap3* knockout.

a Schematic of liver-specific Thrap3 knockout mice; **b** Expression of *Thrap3* in indicated tissues of Thrap3 F/F and Thrap3 LKO were determined by quantitative RT-PCR (n = 6 per group). Values represent means ± SEM. ****P* < 0.001 vs F/F; **c** Expressions of hepatic Thrap3 were determined by western blots.

Supplementary Fig. 3: Expression of genes involved in glycerolipid synthesis and fatty acid uptake.

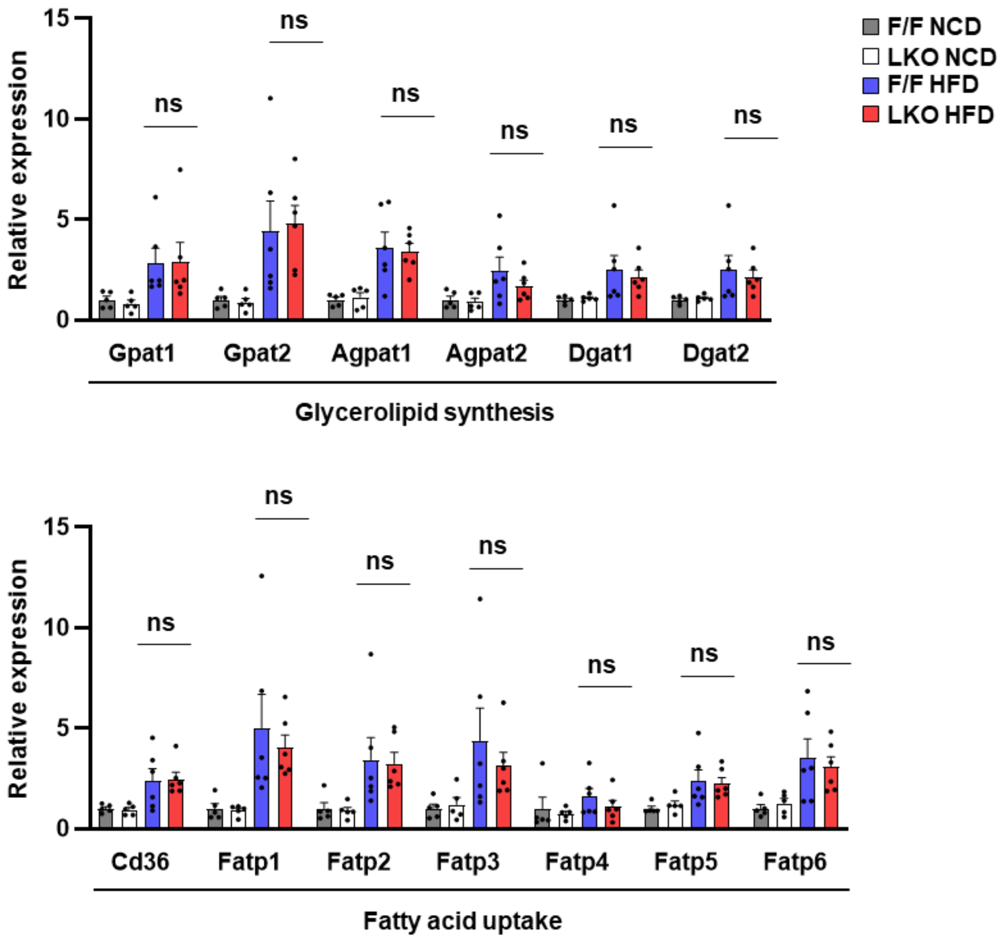

Supplementary Fig. 3: Expression of genes involved in glycerolipid synthesis and fatty acid uptake.

Genes related to glycerolipid synthesis and fatty acid uptake were determined by quantitative RT-PCR. Relative values are normalized to Thrap3 F/F NCD (NCD, n = 5 per group; HFD, n = 6 per group). Values represent means ± SEM. ns, not significant.
